# DNA methylation dynamics during germline development

**DOI:** 10.1111/jipb.13422

**Published:** 2022-12-29

**Authors:** Shengbo He, Xiaoqi Feng

**Affiliations:** ^1^ Guangdong Laboratory for Lingnan Modern Agriculture, College of Agriculture South China Agricultural University Guangzhou 510642 China; ^2^ John Innes Centre, Colney Lane Norwich NR4 7UH UK

**Keywords:** chromatin, DNA methylation, epigenetic reprogramming, germline, small interfering RNA

## Abstract

DNA methylation plays essential homeostatic functions in eukaryotic genomes. In animals, DNA methylation is also developmentally regulated and, in turn, regulates development. In the past two decades, huge research effort has endorsed the understanding that DNA methylation plays a similar role in plant development, especially during sexual reproduction. The power of whole‐genome sequencing and cell isolation techniques, as well as bioinformatics tools, have enabled recent studies to reveal dynamic changes in DNA methylation during germline development. Furthermore, the combination of these technological advances with genetics, developmental biology and cell biology tools has revealed functional methylation reprogramming events that control gene and transposon activities in flowering plant germlines. In this review, we discuss the major advances in our knowledge of DNA methylation dynamics during male and female germline development in flowering plants.

## Graphical abstract

Powered by epigenomic sequencing and cell isolation techniques, recent research has greatly advanced our understanding of plant reproductive epigenetics. This review discusses the major advances in our knowledge of DNA methylation dynamics during plant reproduction, emphasizing methylation reprogramming events that control gene and transposon activities in the germline.

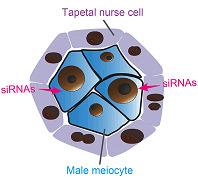

## INTRODUCTION

The addition of a methyl group to the 5th carbon of cytosine, referred to as 5‐methylcytosine, is a prevalent DNA modification that plays essential regulatory roles in eukaryotic genomes (Zemach and Zilberman, 2010; Smith and Meissner, 2013; Zhang et al., 2018). Cytosine methylation in the CG‐dinucleotide context is maintained by DNA methyltransferase 1 (Dnmt1, called MET1 in plants), which methylates hemimethylated CG sites during DNA replication (Law and Jacobsen, 2010). Plant transposable elements (TEs) are also methylated in CHG and CHH contexts (where H is A, C, or T) by two plant‐specific DNA methyltransferases, CHROMOMETHYLASE 3 (CMT3; CHG) and CMT2 (CHH) (Zhang et al., 2018). CMTs preferentially bind to the heterochromatin mark, histone H3 Lys9 dimethylation (H3K9me2), catalyzed by a group of histone methyltransferases, namely SU (var)3‐9 homologue 4/5/6 (SUVH4/5/6), to induce non‐CG methylation, which in turn promotes H3K9me2 mediated by SUVH4/5/6, forming a self‐reinforcing loop (Zhang et al., 2018).

The establishment of *de novo* methylation in all sequence contexts, and maintenance of non‐CG methylation, is catalyzed by plant Dnmt3 homologs (DOMAINS REARRANGED METHYLTRANSFERASE1 and 2, and DRM1 and 2, in *Arabidopsis thaliana*) (Law and Jacobsen, 2010). DRMs function within the small RNA‐directed DNA methylation pathway (RdDM). RdDM comprises two large sets of components responsible for small interfering RNA (siRNA) biogenesis (simplified as the RNA Polymerase IV (Pol IV) pathway) and DNA methylation (the RNA Polymerase V (Pol V) pathway). In the Pol IV pathway, transcripts are produced by a plant‐specific RNA Pol IV, converted into double‐stranded by RNA‐dependent RNA polymerase 2 (RDR2) (Huang et al., 2021), and cleaved by Dicer‐like 3 (DCL3) into 24‐nt siRNAs (Matzke and Mosher, 2014). In the Pol V pathway, the siRNA is loaded into an Argonaute protein‐containing effector complex, which binds to a homologous transcript generated by another plant‐specific RNA polymerase, Pol V, and recruits DRM methyltransferases (Matzke and Mosher, 2014). Pol IV and Pol V preferentially associate with CHG/H methylated DNA, making RdDM a self‐reinforcing pathway in which DNA methylation promotes the generation of methylation‐inducing sRNAs (Kuo et al., 2017; Wendte and Pikaard, 2017).

Loss of DNA methylation can occur passively, by maintenance failure during DNA replications, or actively, via DNA demethylases. In animals and plants, active DNA demethylation requires the excision of 5mC or its derivatives by DNA glycosylases, and the following base excision repair pathway that repairs the DNA with unmethylated cytosine. In animals, 5mC is first oxidated or deaminated before being excised by DNA mismatch repair glycosylases, whereas plant DNA glycosylases directly excise 5mC (Zhang et al., 2018). The involvement of DNA glycosylases in active DNA demethylation was uncovered in *Arabidopsis* through the discovery of REPRESSOR OF SILENCING 1 (ROS1) and its homolog DEMETER (DME) 20 years ago (Choi et al., 2002; Gong et al., 2002). ROS1, DME, DEMETER‐LIKE PROTEIN 2 (DML2) and DML3 are a subfamily of bifunctional DNA glycosylases, which excise 5mC regardless of sequence context (Choi et al., 2002; Gong et al., 2002; Gehring et al., 2006; Penterman et al., 2007; Ortega‐Galisteo et al., 2008; Zhu, 2009). ROS1, DML2 and DML3 are expressed mainly in somatic tissues, while DME is preferentially expressed in the gamete companion cells, i.e., the vegetative cell of pollen and the central cell of female gametophytes (Choi et al., 2002; Gong et al., 2002; Ortega‐Galisteo et al., 2008; Calarco et al., 2012; Ibarra et al., 2012; Park et al., 2017).

DNA methylation patterns are faithfully replicated during cell divisions, thus allowing methylation to exert homeostatic functions during development (Smith and Meissner, 2013; Pikaard and Scheid, 2014). However, in animals and plants, essential methylation reprogramming occurs in the germlines (Seisenberger et al., 2013; Tang et al., 2016; Walker et al., 2018). Mammalian germlines undergo genome‐wide demethylation soon after their specification in the embryo (Seisenberger et al., 2013). This demethylation is crucial for epigenetic resetting, restoration of pluripotency and erasure of parental imprints (Seisenberger et al., 2013; Tang et al., 2016). Subsequently, methylation is re‐established globally, including imprints representative of the sex of the embryo (Seisenberger et al., 2013). Remethylation is mediated by Dnmt3 *de novo* methyltransferases and piwi‐interacting RNAs (piRNAs), a class of siRNAs specifically expressed in gonads (Tóth et al., 2016; Greenberg and Bourc'his, 2019). Impairment of methylation reprogramming reduces male and female fertility (Stewart et al., 2016; Greenberg and Bourc'his, 2019). For example, male meiosis is arrested at the pachytene stage, associated with the derepression of TEs and gene misregulation (Greenberg and Bourc'his, 2019).

**Figure 1:**
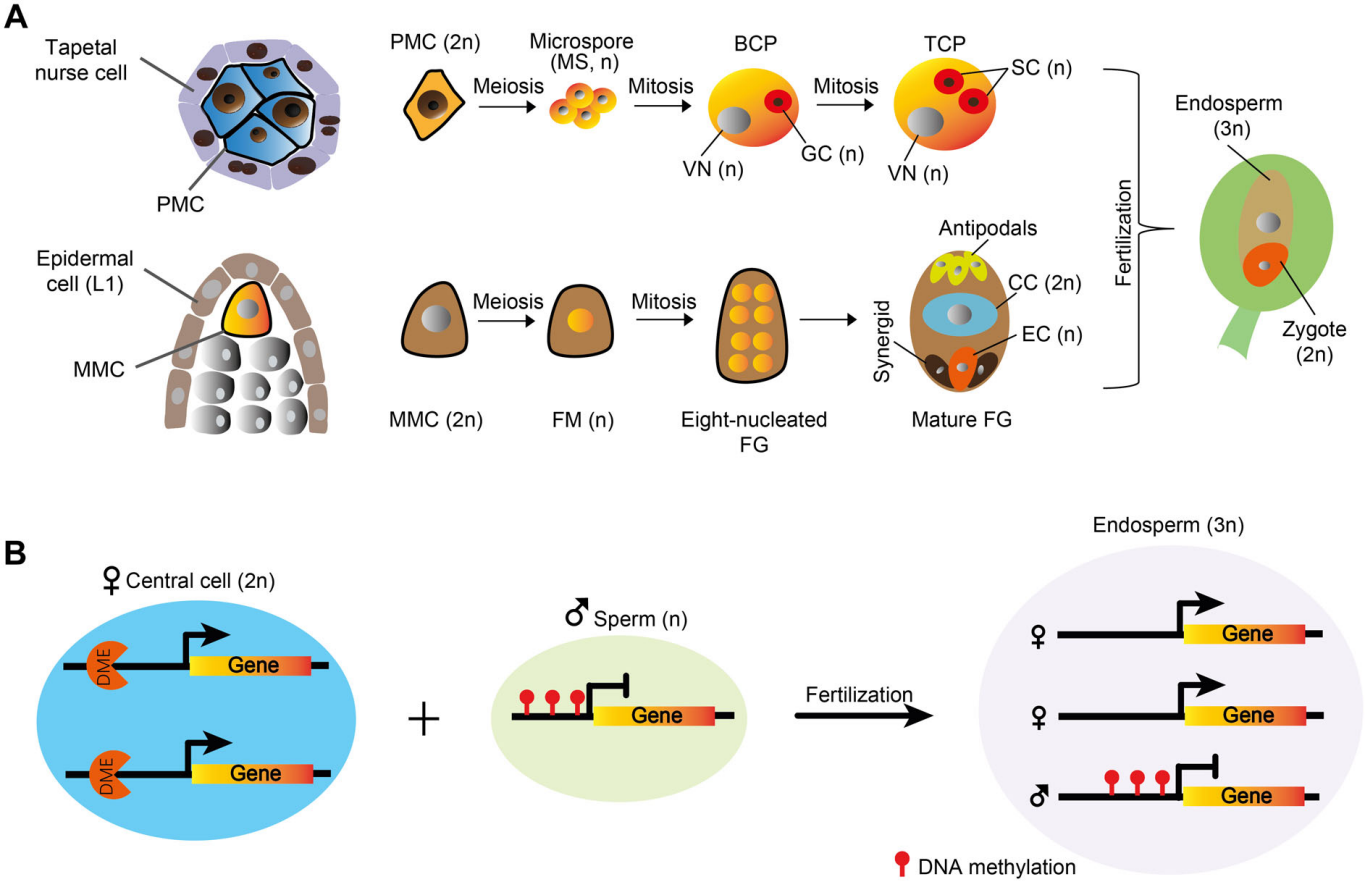
Germline development and double fertilization in plants (**A**) Schematic diagram depicting male and female germline development in *Arabidopsis*. Ploidy levels of cells are indicated. PMC, pollen mother cell; MMC, megaspore mother cell; MS, microspore; FM, functional megaspore; BCP, bicellular pollen; TCP, tricellular pollen; VN, vegetative nucleus; SC, sperm cell; FG, female gametophyte; EC, egg cell; CC, central cell. (**B**) Diagram illustrating the initiation of DNA demethylation at a maternally expressed imprinted gene in the central cell by DME. After fertilization in the endosperm, the paternal allele remains methylated and silenced, whereas the maternal alleles are expressed due to the loss of DNA methylation that occurred earlier in the central cell.

Plants and animals last shared a common unicellular sexual ancestor over a billion years ago (Parfrey et al., 2011). As multicellularity evolved separately in plants and animals, they also adopted distinctive reproductive strategies (Schmidt et al., 2015; Hackenberg and Twell, 2019). Whilst animals usually have a reserved germline sequestered early in development, plants develop the germline from mature somatic cells (Schmidt et al., 2015; Vielle‐Calzada, 2017). Despite this difference, plants and animals convergently evolved specialized nurse cell lineages to nourish the developing germline (Feng et al., 2013). For example, the male germline in *Arabidopsis* initiates as pollen mother cells (also called male meiocytes), which together with their surrounding nurse cells, tapetal cells (collectively called tapetum), descend from a common somatic precursor (Feng et al., 2013; Gómez et al., 2015) (Figure 1A). Enclosed in this essential nurse cell layer, male meiocytes undergo meiosis to generate haploid microspores, each of which divides twice mitotically to give rise to two sperm and a companion vegetative cell in a pollen grain (Hackenberg and Twell, 2019) (Figure 1A). In the *Arabidopsis* ovule, one subepidermal cell differentiates as the female meiocyte (also called the megaspore mother cell), which is surrounded by nucellar cells (Figure 1A). After meiosis, one of the four haploid megaspores survives and continues to divide mitotically into an 8‐nucleated embryo sac, containing a haploid egg cell, a diploid central cell and other accessory cells (Figure 1A). Double fertilization, which is a unique feature of flowering plants, occurs via the fusion of two sperm cells with the egg and central cells, respectively, forming the diploid embryo and triploid endosperm, a nutritious tissue supporting embryo development (Schmidt et al., 2015; Figueiredo and Köhler, 2016) (Figure 1A).

Unlike mammals, plant male and female germlines do not undergo genome‐wide demethylation and remethylation (Kawashima and Berger, 2014). However, dynamic, functional DNA methylation changes take place during germline development. Here we review the progress made in our understanding of these DNA methylation changes, which was initiated and inspired by the discovery of DME 20 years ago.

## ACTIVE DNA DEMETHYLATION IN GAMETE COMPANION CELLS

### DME‐directed DNA demethylation in companion cells

DME was discovered to be essential for the maternal expression of an imprinted gene *MEDEA* (*MEA*) in the endosperm, which is required for seed viability (Choi et al., 2002). Imprinting refers to a phenomenon where genes are expressed in a parent‐of‐origin‐specific fashion (Gehring, 2013; Batista and Köhler, 2020). For example, the Polycomb group gene *MEA* is only expressed from the maternal alleles in the endosperm, whereas the paternal allele is repressed (Grossniklaus et al., 1998; Kinoshita et al., 1999; Kiyosue et al., 1999; Luo et al., 1999). In the endosperm, DME is required for the hypomethylation and expression of the maternal alleles of *MEA*, and other imprinted genes, such as *FWA* and *FIS2* (Choi et al., 2002; Kinoshita et al., 2004; Gehring et al., 2006; Bauer and Fischer, 2011). Bisulfite sequencing of *Arabidopsis* endosperm and embryo revealed DME‐dependent large‐scale maternal DNA hypomethylation in the endosperm that correlates with the expression of imprinted genes, showing that DME‐directed active DNA demethylation lays down the primary imprinting mark in the endosperm (Gehring et al., 2009; Hsieh et al., 2009; Ibarra et al., 2012). This result is further confirmed in the endosperm of other species such as rice and maize, indicating a widely conserved mechanism in flowering plants (Zemach et al., 2010; Xu et al., 2022).

How does DME distinguish between the maternal and paternal genomes in the endosperm? DME expression is not detected in the endosperm but is specifically enriched in the central cell before fertilization (Choi et al., 2002). As the maternal genome of the endosperm is derived from the central cell, a plausible explanation is that active DNA demethylation occurs in the central cell before fertilization (Figure 1B). Owing to the advances in low‐input Next Generation Sequencing (Smallwood et al., 2014) and cell isolation techniques, such as INTACT (Isolation of Nuclei Tagged in specific Cell Types) (Deal and Henikoff, 2011), central cell DNA methylomes were obtained from *Arabidopsis* and rice, demonstrating DNA demethylation indeed occurs in the central cell and is mediated by DME and its rice homolog ROS1a (Choi et al., 2002; Ibarra et al., 2012; Park et al., 2016b) (Figure 1B).

**Figure 2:**
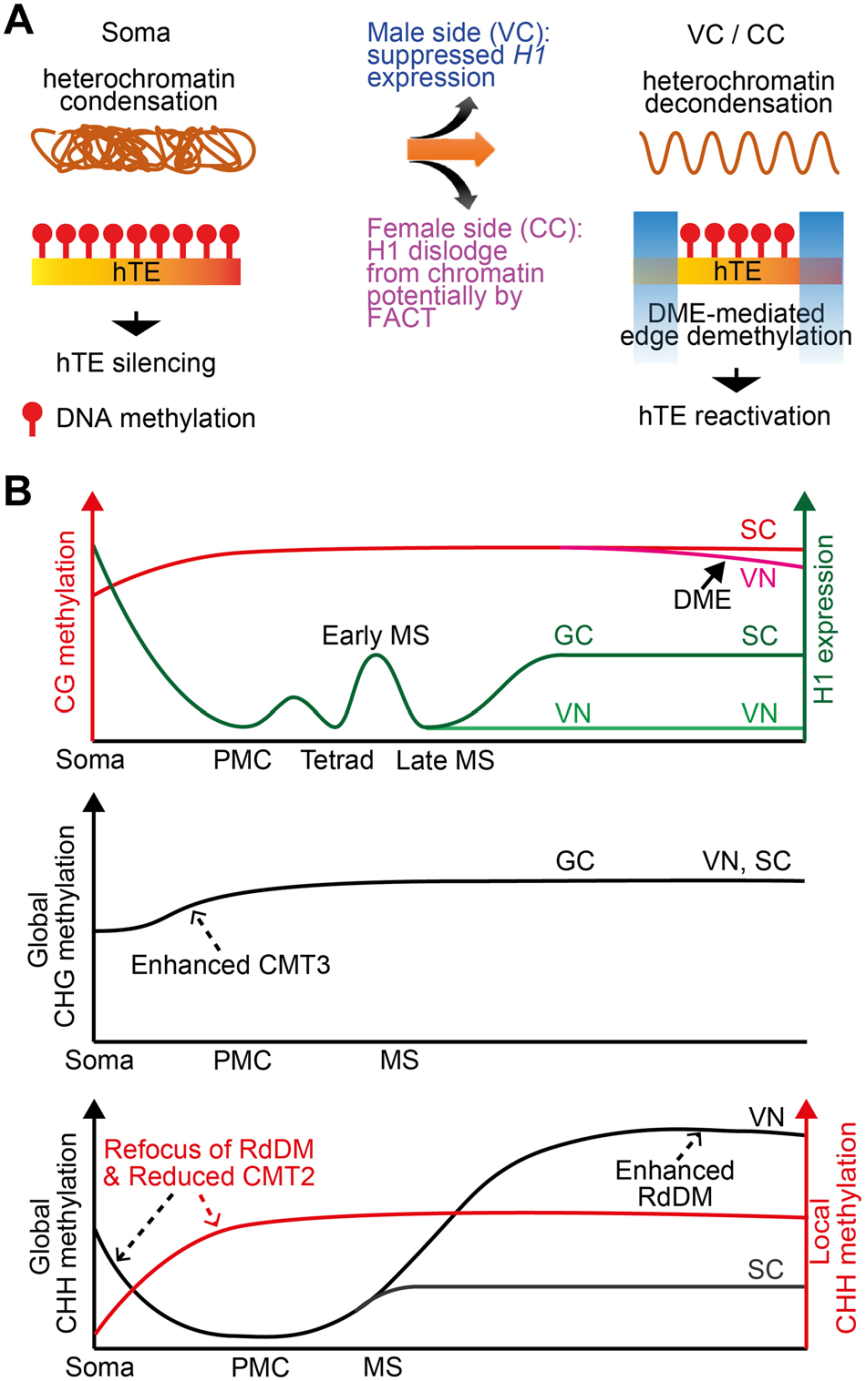
DNA methylation dynamics in the male germline (**A**) Chromatin regulation of DME activity at heterochromatic transposable elements (TEs). In the somatic nucleus, heterochromatin is condensed and heterochromatic TEs (hTEs) are heavily DNA methylated and silenced. In the vegetative cell (VC) and central cell (CC), decondensation of heterochromatin permits the access of DME at the edges of hTEs, causing demethylation and hTE reactivation. Such decondensation is facilitated by H1 depletion, which naturally happens in the VC and is possibly mediated by FACT in the CC. (**B**) Schematic diagram illustrating DNA methylation changes during male germline development in *Arabidopsis*. We hypothesize that reduced H1 (shown in green) in the germline (compared to soma) contributes to enhanced CG methylation in the germline. Other involved mechanisms are indicated by arrows, with dashed arrows showing potential mechanisms that require further testing. Abbreviations are used as in Figure 1A.

Besides the central cell, DME is also expressed in the sperm companion cell, the vegetative cell (Figure 2A) (Schoft et al., 2011; Park et al., 2017). DME‐regulated imprinted genes, such as *MEA* and *FWA*, are demethylated and transcribed in the vegetative cell (Schoft et al., 2011), suggesting active DNA demethylation also occurs in vegetative cells. Indeed, vegetative cells of *Arabidopsis* and rice show prominent local hypomethylation in all sequence contexts compared to sperm, and this hypomethylation requires DME in *Arabidopsis*, or ROS1a in rice (Figure 2A, B) (Calarco et al., 2012; Ibarra et al., 2012; Kim et al., 2019).

Besides gene regulation in the endosperm and vegetative cell (Ibarra et al., 2012; Borg et al., 2021; Khouider et al., 2021), DME‐mediated active demethylation has been proposed to serve genome defense functions. In the vegetative cell, 21‐nucleotide (nt) siRNAs were found to accumulate at reactivated TEs (Slotkin et al., 2009). These TEs are hypermethylated and silenced in the sperm, suggesting that vegetative‐cell‐derived siRNAs move into the sperm cell and reinforce TE silencing (Slotkin et al., 2009). The ability of siRNAs to travel from vegetative cells to sperm is supported by experiments showing the silencing of sperm‐expressed Green Fluorescent Protein by vegetative‐cell‐expressed siRNAs (Martínez et al., 2016). Furthermore, DME‐directed active DNA demethylation at thousands of TEs in the vegetative cell is required for CHH methylation of cognate transposons in sperm (Ibarra et al., 2012). The genetic requirement of a demethylase for DNA hypermethylation shows that siRNA movement is at least a straightforward explanation for the reinforced TE methylation in sperm. However, siRNA movement between vegetative and sperm cells is still under some debate due to the lack of direct evidence and demonstrated cytoplasmic connections between the vegetative and sperm cells (Kawashima and Berger, 2014). On the female side, siRNA expression in the central cell has been shown to induce post‐transcriptional silencing in the egg cell (Ibarra et al., 2012) and there are well‐documented cytoplasmic connections between these cells (Han et al., 2000; Erdmann et al., 2017). Therefore, epigenetic reprogramming in the egg cell by central cell siRNAs is highly plausible (Feng et al., 2013).

### Companion cell chromatin decondensation is required for DME activity

It is yet unclear how DME/ROS1a is recruited to genomic targets but this process is sensitive to the chromatin environment (Feng et al., 2013). DME preferentially targets small AT‐rich euchromatic TEs and the edges of long heterochromatic TEs in both vegetative and central cells (Ibarra et al., 2012). Interestingly, although DME demethylates approximately 10,000 loci similarly in vegetative cells and maternal endosperm, only half of the male and female targets overlap (Ibarra et al., 2012). This likely reflects different chromatin environments in male and female companion cells (Feng et al., 2013). Indeed, although the central and vegetative cells both exhibit highly decondensed chromatin, there are differences in their chromatin configurations (Baroux and Autran, 2015; Borg and Berger, 2015) (Figure 2A). In the vegetative cell, linker histone H1 is depleted, which contributes to the decondensation of heterochromatin and DME's access to heterochromatic regions (He et al., 2019). Ectopic expression of H1 in the vegetative cell causes hypermethylation and suppression of DME‐activated heterochromatic TEs (He et al., 2019). In the endosperm, DME's access to heterochromatic TEs requires FACT (FAcilitates Chromatin Transactions), a highly conserved histone chaperone complex important for nucleosome assembly during transcription and DNA replication (Frost et al., 2018). *h1* mutations abolish the requirement of FACT at about 10% of DME targets in the endosperm (Frost et al., 2018), suggesting that FACT acts to enhance DME accessibility in the central cell by counteracting H1 (Figure 2A). The SSRP1 subunit of the FACT complex belongs to a superfamily of High Mobility Group proteins, which tend to compete with H1 for chromatin binding in a dose‐dependent manner (Postnikov and Bustin, 2016). In the central cell, SSRP1 likely triggers H1 dislodge from chromatin, thereby permitting DME access to the target sites (Frost et al., 2018) (Figure 2A). Consistent with the observed H1 depletion in the vegetative cell (He et al., 2019), FACT is not required for DME activity in the vegetative cell (Frost et al., 2018). These results demonstrate that male and female companion cells undergo distinct chromatin decondensation, contributed by multiple factors, and such decondensation is important for large‐scale DME‐mediated DNA demethylation.

## ROBUST DNA METHYLATION MAINTENANCE IN GERMLINES

The plant germline, by strict definition, refers to the cells that exclusively produce gametes. Thus, the male germline starts as the generative cell that divides into sperm cells in the pollen or pollen tube, whereas the female germline refers to the egg cell (Figure 1A). In comparative studies with animals, it is helpful to define germlines more broadly, to include the sporophytic cells that undergo meiosis to produce the gametophytes (equivalent to primordial germ cells in animals), i.e., the pollen and megaspore mother cells (PMC and MMC, respectively; Figure 1A). PMC and MMC undergo meiosis to give rise to haploid microspores and megaspores, respectively, which divide mitotically to give rise to gametes and their companion cells in the gametophytes (Figure 1A).

Comparisons of DNA methylomes between the *Arabidopsis* male germ cells and somatic cells/tissues showed dynamic methylation changes during germline development (Hsieh et al., 2016; Walker et al., 2018). The PMC (male meiocyte), microspore and sperm have substantially higher and lower methylation, respectively, in the CHG and CHH contexts compared to somatic tissues, suggesting generally enhanced CMT3 and reduced CMT2 activities (Calarco et al., 2012; Hsieh et al., 2016; Walker et al., 2018) (Figure 2B). The vegetative cell has similar CHG methylation to other male germ cells but much higher CHH methylation (Figure 2B), owing to elevated RdDM activity, especially at heterochromatic regions (Hsieh et al., 2016). Global CG methylation is substantially higher in the germline than in somatic cells (Hsieh et al., 2016; Walker et al., 2018) (Figure 2B). In the meiocyte, microspore, and sperm cell (and to a slightly lesser extent the vegetative cell, due to DME‐mediated demethylation), fractional CG methylation reaches almost 100% (Hsieh et al., 2016; Walker et al., 2018), indicating enhanced CG methylation in the germline (Figure 2B). Similarly, robust CG methylation is observed in female central cells in *Arabidopsis* and egg cells in rice (Park et al., 2016b), indicating a conserved phenomenon in the male and female germlines of flowering plants. Improved CG methylation in the germlines is likely caused by increased efficiency of CG methylation maintenance (via MET1), as none of the other methyltransferases (DRM, CMT2 and CMT3) affect robust CG methylation in germ cells (Hsieh et al., 2016). It is yet unclear how CG methylation maintenance is enhanced, however, reduced H1 observed in germ cells, such as the sperm, in comparison to H1 levels in soma, may contribute to easier access of MET1 to the DNA (Hsieh et al., 2016) (Figure 2B). In flowering plants, heredity is confined to a small group of cells that undergo limited numbers of cell divisions: the germlines produce gametes, which fuse into a zygote; the zygote then divides into an early embryo with shoot apical meristematic cells that later give rise to male and female germlines (Figure 1A). It is estimated that this extended ‘germline' goes through approximately 30‐40 cell divisions in each life cycle in *Arabidopsis* (Watson et al., 2016). It is thus conceivable that enhanced methylation maintenance efficiency in this extended ‘germline’ contributes to the accurate inheritance of methylation patterns across generations in flowering plants.

## DNA METHYLATION REPROGRAMMING IN THE MALE GERMLINE

### *de novo* methylation of genes in the germline

Although CG methylation is globally high in the germlines, CHH methylation at most TEs is surprisingly low in sperm cells (Calarco et al., 2012; Ibarra et al., 2012). This was proposed to be caused by defective methylation maintenance during meiosis (Calarco et al., 2012). However, this notion is counterintuitive because TE silencing is especially important in cells that mediate inheritance. A careful comparison of the male meiocyte methylome to those of microspores and sperm revealed that meiocytes have the lowest overall level of CHH methylation (Walker et al., 2018) (Figure 2B). Thus, CHH methylation is not suspended during meiosis, but rather increases in microspores and sperm (Walker et al., 2018) (Figure 2B).

**Figure 3:**
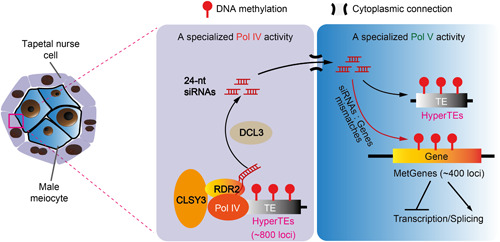
Molecular mechanism of male germline DNA methylation reprogramming DNA methylation reprogramming in Arabidopsis male germline is driven by tapetal 24‐nt siRNAs, which are transcribed from HyperTE loci via the Pol IV machinery recruited by the tapetum‐specific CLSY3. These tapetal siRNAs act *in trans* at both cellular and locus levels: they move into male meiocytes possibly through plasmodesmata, and target methylation at HyperTE loci with perfect sequence homologies as well as MetGene loci that have similar sequences to HyperTEs. This methylation reprogramming has dual functions in silencing TEs in the germline and regulating gene expression and splicing in the meiocyte. The diagram is adapted from Long et al. (2021). TEs, transposable elements.

This gradual change of CHH methylation during male germline development reflects reduced CMT2 activity and a re‐focus of RdDM activity in these germ cells (Figure 2B). Indeed, RdDM, which almost exclusively methylates TEs in somatic tissues, acquires a special activity to target genes in the entire male germline, including meiocytes, microspores, vegetative and sperm cells (Walker et al., 2018). This manifests in loci that are CHG/H methylated exclusively in the male germline, which are called germline‐specific methylated loci (~400 loci; mostly genes and hence simplified as MetGenes) (2, 3). Despite the generally low level of CHH methylation at somatic RdDM targets, some of these loci exhibit CHG/H hypermethylation in all male germ cells (~800 loci; mostly transposons and hence simplified as HyperTEs) (Figure 3). Hypermethylation at MetGenes and HyperTEs is completely abolished in RdDM mutants, such as *pol iv*, *rdr2*, and *drm1drm2* double mutants, demonstrating that this methylation reprogramming is catalyzed by RdDM (Walker et al., 2018).

Consistent with the observed methylation patterns, male meiocytes have a distinctive siRNA profile from somatic tissues, with the vast majority (over 90%) of 24‐nt siRNAs being concentrated at HyperTEs (Long et al., 2021). A rough gauge of absolute quantities of siRNAs using total microRNA levels shows that the levels of 24‐nt siRNAs at canonical RdDM loci are similar in meiocytes and soma. Therefore, the meiocyte *de novo* methylome seems to be largely driven by an overwhelming increase of siRNAs produced from a few HyperTEs. Interestingly, MetGenes share sequence similarities with HyperTEs but are associated with few perfectly matching 24‐nt siRNAs in meiocytes. When up to three mismatches are permitted, siRNAs, which perfectly map to HyperTEs, then find MetGenes as their closest genomic targets (Long et al., 2021), suggesting that MetGemes are off‐targets of HyperTE‐derived siRNAs. Indeed, in CRISPR lines with the predicted source HyperTEs deleted, MetGene methylation is lost (Long et al., 2021). Therefore, DNA methylation at MetGenes is induced *in trans* by HyperTE‐produced siRNAs (Figure 3).

### Germline methylation reprogramming controls gene and TE activities

It is well known that DNA methylation levels fluctuate during plant development and under different environmental conditions, especially CHH methylation (Dubin et al., 2015; Kawakatsu et al., 2016a, 2016b). Therefore, in this review, we only refer to distinctive DNA methylation changes that have demonstrated biological functions as DNA methylation reprogramming. The *de novo* methylation at MetGenes in the male germline fits this criterion: this methylation regulates gene expression in male meiocytes and facilitates the splicing of a key meiotic gene, *MULTIPOLAR SPINDLE 1* (*MPS1*; also known as *PUTATIVE RECOMBINATION INITIATION DEFECTS 2*, *PRD2*), thereby promoting meiosis (Walker et al., 2018). Therefore, this methylation reprogramming creates a cell‐lineage‐specific epigenetic signature that controls gene expression and cellular function.

The finding that genic methylation in the male germline is targeted by TE‐produced siRNAs suggests that the germline gene regulatory functions of RdDM have evolved from the pathway's main TE silencing activity. Supportive of this idea, a Gypsy retrotransposon, *GP1*, was found to be suppressed by germline RdDM (Long et al., 2021). Loss of RdDM does not activate *GP1* in somatic tissues, whereas DNA methylation of the LTRs requires RdDM in the soma and germline (Long et al., 2021). This suggests that *GP1* specifically targets expression in reproductive cells, possibly by exploiting transcription factors specific to these cell types. This may be advantageous for the transposon, as expression in somatic tissues would not contribute to TE inheritance but could trigger silencing via systemic RdDM (Dunoyer et al., 2007; Smith et al., 2007; Molnar et al., 2010; Melnyk et al., 2011; Lewsey et al., 2016). The broad‐targeting capability of RdDM in meiocytes may have evolved to counteract such TEs.

### Germline methylation reprogramming is driven by tapetal siRNAs

Given the self‐reinforcing nature of RdDM, the lack of perfect‐matching 24‐nt siRNAs to MetGenes suggests that siRNA biogenesis (i.e., the Pol IV branch of the RdDM pathway) is suppressed in male meiocytes. As meiocytes are completely enclosed by the tapetal cells (Figure 1A), which share extensive plasmodesmata with meiocytes during early meiosis (Mamun et al., 2005; Sager and Lee, 2014), the tapetum is a plausible source of 24‐nt siRNAs. Indeed, tapetal cells have an siRNA profile resembling meiocytes, and are hypermethylated at HyperTEs (Long et al., 2021). Further, genetic mosaic lines with siRNA biogenesis ability confined to the tapetum, *pA9::RDR2 rdr2* (*pA9*, a tapetum‐specific promoter), show fully restored MetGene (and HyperTE) methylation in both meiocytes and sperm cells, demonstrating that tapetal siRNAs are sufficient to induce germline methylation reprogramming (Long et al., 2021).

How are large quantities of siRNAs produced from HyperTEs in the tapetum? Recent studies found a family of putative chromatin remodelers, CLASSY (CLSY) 1‐4, exhibit cell/tissue‐specific expression patterns and determine siRNA production at different genomic loci via the recruitment of Pol IV (Long et al., 2021; Zhou et al., 2022b). CLSY1 and 2 are the predominant homologs expressed in somatic tissues with little expression in reproductive organs (Smith et al., 2007; Zhou et al., 2018; Zhou et al., 2022b), however, CLSY3 is expressed specifically in male and female reproductive organs, while CLSY4 is mainly expressed in male meiocytes and to a lesser extent in ovules (Long et al., 2021; Zhou et al., 2022b). In the anther, CLSY3 is specific to the tapetum and responsible for HyperTE‐associated siRNAs and MetGene methylation in meiocytes (Long et al., 2021). Therefore, the vast majority of meiocyte 24‐nt siRNAs, if not all, are derived from the tapetum (Figure 3). The Pol IV pathway may be suppressed in meiocytes because siRNA production, which involves transcription, carries the risk of TE activation. Biogenesis of siRNAs might be carried out more safely by the short‐lived tapetum.

Remarkably, tapetal siRNAs can induce DNA methylation not only in meiocytes, but throughout the male germline. The mutation of *CLSY3* abolishes MetGene methylation in sperm, and the *pA9::RDR2 rdr2* mosaic lines with siRNA biogenesis confined to the tapetum restore not only sperm MetGene methylation, but also methylation at canonical RdDM loci and *GP1* silencing (Long et al., 2021). As direct siRNA movement between pollen and tapetum is hampered by the pollen wall (Gómez et al., 2015), tapetal siRNAs most likely influence sperm via inheritance from meiocytes. Previously, siRNAs from the pollen vegetative cell were shown to reinforce TE methylation in sperm (Slotkin et al., 2009; Ibarra et al., 2012). The sperm methylation landscape is therefore likely determined by two waves of exogenous siRNAs, first from the tapetum and another from the vegetative cell. The tapetum plays a central role in this reprogramming process, as tapetal siRNAs are required to establish MetGene methylation and are competent to drive the full spectrum of sperm RdDM.

Tapetal cells are descendants of the same progenitor cell as meiocytes and specialize in transporting biological materials to the meiocytes (Feng et al., 2013). Thus, they are ideal for genome surveillance of TEs and the production of siRNAs. In meiocytes, the Pol V pathway is tuned more aggressively than in somatic tissues, where siRNAs only target methylation at perfectly matching sequences. The hypersensitivity of the meiocyte Pol V pathway allows the methylation of cognate TEs that produce siRNAs in the tapetum and ones with similar sequences. This hypersensitivity also causes methylation of genes bearing similar sequences in meiocytes (Figure 3), which potentiates transcriptional regulation and hence the selection of beneficial functions during evolution (Walker et al., 2018).

### Tapetal siRNAs closely resemble phasiRNAs

24‐nt siRNAs responsible for male germline methylation reprogramming accumulate in *Arabidopsis* tapetum during the prophase of meiosis I. Other plant species accumulate 24‐nt phased siRNAs (phasiRNAs) in the tapetum at a similar developmental stage (Zhai et al., 2015; Fei et al., 2016; Kakrana et al., 2018; Ono et al., 2018; Xia et al., 2019). The phasiRNA clusters of monocot species such as maize and rice differ in their biogenesis from tapetal siRNAs, as they are transcribed by Pol II and are processed by a monocot‐specific family of DCL nucleases (DCL5) (Johnson et al., 2009; Song et al., 2012; Xia et al., 2019; Teng et al., 2020). Nonetheless, like *Arabidopsis* tapetal siRNAs, phasiRNAs are transcribed from hundreds of loci and are highly abundant, accounting for 64% of all 24‐nt siRNAs in maize anthers (Johnson et al., 2009; Song et al., 2012; Komiya et al., 2014; Zhai et al., 2015). Comparable to the importance of siRNAs for meiotic progression (Walker et al., 2018), phasiRNAs are important for fertility in maize and rice (Nan et al., 2016; Ono et al., 2018; Liu et al., 2020; Teng et al., 2020). How 24‐nt phasiRNAs function remains unknown due to the lack of perfectly matching mRNA targets (Dukowic‐Schulze et al., 2016; Zhang et al., 2021). However, recent studies indicate striking similarities between phasiRNAs and *Arabidopsis* tapetal siRNAs. First, like *Arabidopsis* tapetal siRNAs, phasiRNAs are also suggested to be synthesized in the tapetum and transported into meiocytes (Ono et al., 2018; Liu et al., 2020; Zhou et al., 2022a). Second, 24‐nt phasiRNAs also likely direct DNA methylation, as their source loci exhibit significantly reduced CHH methylation in mutants defective in phasiRNA biogenesis (Zhang et al., 2021). These similarities suggest that 24‐nt phasiRNAs might induce DNA methylation *in trans* at genic loci with reduced sequence homology in male meiocytes, like *Arabidopsis* tapetal 24‐nt siRNAs, an exciting hypothesis to be tested for understanding why 24‐nt phasiRNAs are important for fertility. This molecular understanding will undoubtedly facilitate the development of future strategies to manipulate male fertility and sustain crop yields.

### Evolutionary convergence of the tapetal siRNA and animal piRNA pathways

Tapetal siRNAs bear strong similarities to metazoan piRNAs in terms of biogenesis and function. Like piRNAs, tapetal siRNAs are important for fertility (Walker et al., 2018), are specifically enriched in reproductive cells, and are capable of silencing TEs (Walker et al., 2018; Long et al., 2021). siRNAs are produced in tapetal nurse cells and transported into meiocytes, like *Drosophila* piRNAs that are transported from nurse cells into oocytes (Tóth et al., 2016). The ability of tapetal siRNAs from TE clusters to regulate mismatched genes *in trans* is similar to the described silencing of male genes by a piRNA transcribed from repeats on the silkworm female sex chromosome (Kiuchi et al., 2014). Intriguingly, mammalian piRNAs are enriched in pachytene spermatocytes (the same developmental stage as tapetal siRNAs) and lack perfectly matched mRNA targets (Ernst et al., 2017; Ozata et al., 2019). It has been shown that mouse pachytene piRNAs target and post‐transcriptionally regulate mismatched genes (Rojas‐Ríos and Simonelig, 2018; Dai et al., 2019), as do piRNAs in *Drosophila* and *C. elegans* (Rojas‐Ríos and Simonelig, 2018; Ozata et al., 2019). The recurrence of broad targeting competence predicts a general ability of piRNAs and tapetal siRNAs to regulate genes as well as TEs. Overall, the many similarities between piRNAs and tapetal siRNAs indicate that gametogenesis in plants and animals requires specialized small RNA pathways to control TEs and preserve genome integrity, and these pathways have evolved to regulate gene activity and fertility.

## DNA METHYLATION REPROGRAMMING IN THE FEMALE GERMLINE

Female germ cells in flowering plants are much fewer in number than male cells, and are deeply embedded in maternal tissues, making them less accessible for DNA methylation studies. The female germline initiates as MMCs that develop from subepidermal (L2) cells in developing ovule primordia (Figure 1A). Laser‐assisted microdissection has been successfully used to isolate MMCs from *Arabidopsis*, and thereby obtain the MMC transcriptome (Schmidt et al., 2011). Live‐imaging sensors have been developed using fluorescent fusion proteins that contain methylation binding domains, such as the methyl‐CpG‐binding domain (MBD; binds CG methylation) and SET and RING finger‐associated domain (SRA; binds non‐CG methylation) (Ingouff et al., 2017). Using these sensors, CG methylation was shown to be stable during MMC formation, whereas CHH methylation became undetectable in the MMCs (Ingouff et al., 2017). Although no MMC methylome data have been published yet, there are several lines of evidence indicating that DNA methylation reprogramming might also occur in female germlines, in which epigenetic silencing is important for cell fate and function.

Normally in each ovule, only one L2 cell acquires MMC fate and the capability to undergo meiosis (Figure 1A). However, in mutants of RdDM or 21‐22 nt trans‐acting siRNA (tasiRNA) pathways, multiple L2 cells adopt MMC fate (Olmedo‐Monfil et al., 2010; Su et al., 2017; Su et al., 2020), suggesting RNA interference is important for suppressing MMC fate in companion L2 cells. Interestingly, these siRNAs are suggested to be produced by the MMC‐adjacent epidermal (L1) cells, as several key components of the RdDM and tasiRNA pathways, such as AGO9, TEX1 and SGS3, are specifically expressed in L1 cells. Elegant genetic analyses show that L1‐derived tasiRNAs suppress MMC fate in L2 cells via the repression of *Auxin Response Factor 3* (*ARF3*) (Su et al., 2017; Su et al., 2020). The molecular mechanism by which RdDM affects MMC differentiation is yet unclear. Adding to the complexity, mutations in other DNA methyltransferases, such as MET1 (Li et al., 2017) and the maize homolog of CMT3 (Garcia‐Aguilar et al., 2010), also cause a similar supernumerary MMC phenotype. Therefore, it seems that the epigenetic state of L2 cells is critical for MMC differentiation. The supernumerary MMC phenotype mimics an important breeding strategy, apomixis, which refers to the generation of clonal seeds via asexual reproduction. Bypassing meiosis and fertilization, apomixis has the potential to fix desired genetic combinations and hence hybrid vigor in important crop varieties. Understanding the epigenetic regulation of MMC differentiation holds promise for engineering apomixis in crops.

DNA methylation reprogramming in the *Arabidopsis* male germline is driven by large quantities of 24‐nt siRNAs transcribed from a few hundred source loci in the tapetum, mostly overlapping TEs (Long et al., 2021). Similarly, a small number of loci, called siren loci, were found to produce highly abundant 24‐nt, RdDM‐associated siRNAs in *Arabidopsis* and *Brassica rapa* ovules (Grover et al., 2020; Zhou et al., 2022b). Resembling tapetal siRNAs, the specific expression of CLSY3 is responsible for the production of siren siRNAs in *Arabidopsis* ovules (Zhou et al., 2022b). Furthermore, siRNAs from siren loci were suggested to induce methylation at genes with similar but not identical sequences *in trans* and thereby regulate gene expression in *Brassica* ovules (Burgess et al., 2022). CLSY3 is highly expressed in the early ovary and ovules (Long et al., 2021; Zhou et al., 2022b), and it is yet unclear which cell types in the ovule express these siRNAs and are subject to *trans*‐induced methylation. However, a recent study of rice egg and zygote siRNA landscapes shows that the egg cell accumulates abundant 24‐nt siRNAs from siren loci that are distinct from the source loci in sperm (Li et al., 2022), indicating that DNA methylation reprogramming occurs in the female germline, at distinct loci from those in the male germline, and methylation reprogramming is likely a conserved phenomenon among flowering plants.

## CONCLUSIONS AND PERSPECTIVES

Significant progress in reproductive epigenetics has been made in the past twenty years. Advances in cell isolation and low‐input epigenomic sequencing techniques have revealed the landscape of DNA methylation dynamics during flowering plant germline development, especially in the male germline owing to the relative ease in accessing large quantities of male germ cells (Figure 2B). As discussed in this review, besides global fluctuations of DNA methylation in germ cells, there are two waves of methylation reprogramming that occur at specific loci and have demonstrated biological functions. The first wave of reprogramming is directed by an overwhelming population of 24‐nt siRNAs produced from only hundreds of source loci in the tapetum (Long et al., 2021). These tapetal siRNAs target DNA methylation at transposons and genes with reduced sequence homologies in *Arabidopsis* male meiocytes, and through this regulate gene expression and meiosis (Walker et al., 2018; Long et al., 2021). Although we do not yet have methylome data from female meiocytes, data discussed here point to a similar methylation reprogramming in the female germline. Another wave of reprogramming occurs in the male and female gamete companion cells, respectively the central and vegetative cells, where DME drives large‐scale DNA demethylation (Calarco et al., 2012; Ibarra et al., 2012; Park et al., 2016b). Companion cell demethylation plays an essential role in setting up the primary imprinting mark in the endosperm, and activates TEs that potentially serve to generate siRNAs to reinforce genome integrity in the gametes (1, 2).

Future innovations in cell/nucleus isolation techniques will power further understanding of germline DNA methylation reprogramming, especially in female germlines where we currently have little data. Protoplast preparation and fluorescence‐activated cell sorting (FACS) have been exploited to isolate plant male germ cells using cell‐type‐specific fluorescent labelling or different cell properties such as cell size, autofluorescence and DNA density (Slotkin et al., 2009; Borges et al., 2012; Calarco et al., 2012; Ibarra et al., 2012; Hsieh et al., 2016; Santos et al., 2017; Chang et al., 2018; Walker et al., 2018; He et al., 2019; Zheng and Gehring, 2019; Buttress et al., 2022). For some species or particular cell types, microdissection serves well (Sprunck et al., 2005; Schmidt et al., 2011; Park et al., 2016b; Flores‐Tornero et al., 2019; Zhao et al., 2019; Jiang et al., 2020), especially now only a few cells are required for DNA methylome sequencing (Smallwood et al., 2014). Female reproductive cells are much more challenging to isolate as not only are they far fewer in number, but also deeply embedded in maternal tissues. Adding to the complexity, emasculation or male sterile lines are often required to prevent the fertilization of female cells. INTACT has been successfully employed to assess the methylomes of central cells and the endosperm in *Arabidopsis* (Park et al., 2016b; Moreno‐Romero et al., 2017; Del Toro‐De León and Köhler, 2019). Isolating nuclei through cell‐specific affinity labelling, INTACT, is an efficient, scalable method that holds great promise for the future (Park et al., 2016a; Moreno‐Romero et al., 2017; Del Toro‐De León and Köhler, 2019).

The next challenge is to gain a better mechanistic understanding of DNA methylation changes during germline development. This understanding will not only deepen our knowledge of plant reproduction, but also provide valuable mechanistic insights into DNA methylation, especially regarding how these mechanisms operate at a cellular level. Another important and challenging question to address regarding these mechanisms concerns the site‐specificity of DNA methylation reprogramming processes, i.e., why reprogramming occurs at specific genomic loci? In this review, we suggest that the local chromatin environment plays an important role in determining such specificity. The evaluation and validation of this idea through an enhanced understanding of germ cell chromatin landscapes will be a leap forward in understanding germline epigenetics. Finally, it will be vital to combine 'omics with in‐depth functional studies at specific loci to discern the epigenetic regulation of reproductive development. As epigenetic pathways are highly conserved between *Arabidopsis* and crops, we expect the mechanistic understanding gained from *Arabidopsis* to direct investigations into crops. Given the availability of crop genome sequences and genome/epigenome editing tools, these investigations hold great potential for sustaining crop fertility under climate change and engineering fertility to facilitate crop breeding.

## CONFLICTS OF INTEREST

The authors declare that they have no conflicts of interest associated with this work.

## AUTHOR CONTRIBUTIONS

S.H. and X.F. conceived the article, searched the literature, and wrote the manuscript.

## References

[jipb13422-bib-0001] Baroux, C. , and Autran, D. (2015). Chromatin dynamics during cellular differentiation in the female reproductive lineage of flowering plants. Plant J. 83: 160–176. 26031902 10.1111/tpj.12890 PMC4502977

[jipb13422-bib-0002] Batista, R.A. , and Köhler, C. (2020). Genomic imprinting in plants—revisiting existing models. Genes Dev. 34: 24–36. 31896690 10.1101/gad.332924.119 PMC6938664

[jipb13422-bib-0003] Bauer, M.J. , and Fischer, R.L. (2011). Genome demethylation and imprinting in the endosperm. Curr. Opin. Plant Biol. 14: 162–167. 21435940 10.1016/j.pbi.2011.02.006 PMC3082360

[jipb13422-bib-0004] Borg, M. , and Berger, F. (2015). Chromatin remodelling during male gametophyte development. Plant J. 83: 177–188. 25892182 10.1111/tpj.12856

[jipb13422-bib-0005] Borg, M. , Papareddy, R.K. , Dombey, R. , Axelsson, E. , Nodine, M.D. , Twell, D. , and Berger, F. (2021). Epigenetic reprogramming rewires transcription during the alternation of generations in *Arabidopsis* . eLife 10: e61894. 33491647 10.7554/eLife.61894 PMC7920552

[jipb13422-bib-0006] Borges, F. , Gardner, R. , Lopes, T. , Calarco, J.P. , Boavida, L.C. , Slotkin, R.K. , Martienssen, R.A. , and Becker, J.D. (2012). FACS‐based purification of *Arabidopsis* microspores, sperm cells and vegetative nuclei. Plant Methods 8: 44. 23075219 10.1186/1746-4811-8-44 PMC3502443

[jipb13422-bib-0007] Burgess, D. , Chow, H.T. , Grover, J.W. , Freeling, M. , and Mosher, R.A. (2022). Ovule siRNAs methylate protein‐coding genes in trans. Plant Cell 34: 3647–3664. 35781738 10.1093/plcell/koac197 PMC9516104

[jipb13422-bib-0008] Buttress, T. , He, S. , Wang, L. , Zhou, S. , Saalbach, G. , Vickers, M. , Li, G. , Li, P. , and Feng, X. (2022). Histone H2B.8 compacts flowering plant sperm through chromatin phase separation. Nature 611: 614–622. 36323776 10.1038/s41586-022-05386-6 PMC9668745

[jipb13422-bib-0009] Calarco, J.P. , Borges, F. , Donoghue, M.T.A. , Van Ex, F. , Jullien, P.E. , Lopes, T. , Gardner, R. , Berger, F. , Feijó, J.A. , Becker, J.D. , and Martienssen, R.A. (2012). Reprogramming of DNA methylation in pollen guides epigenetic inheritance via small RNA. Cell 151: 194–205. 23000270 10.1016/j.cell.2012.09.001 PMC3697483

[jipb13422-bib-0010] Chang, P. , Tseng, Y.‐F. , Chen, P.‐Y. , and Wang, C.‐J.R. (2018). Using flow cytometry to isolate Maize meiocytes for next generation sequencing: A time and labor efficient method. Curr. Protoc. Plant Biol 3: e20068. 29927118 10.1002/cppb.20068

[jipb13422-bib-0011] Choi, Y. , Gehring, M. , Johnson, L. , Hannon, M. , Harada, J.J. , Goldberg, R.B. , Jacobsen, S.E. , and Fischer, R.L. (2002). DEMETER, a DNA glycosylase domain protein, is required for endosperm gene imprinting and seed viability in *Arabidopsis* . Cell 110: 33–42. 12150995 10.1016/s0092-8674(02)00807-3

[jipb13422-bib-0012] Dai, P. , Wang, X. , Gou, L.‐T. , Li, Z.‐T. , Wen, Z. , Chen, Z.‐G. , Hua, M.‐M. , Zhong, A. , Wang, L. , Su, H. , Wan, H. , Qian, K. , Liao, L. , Li, J. , Tian, B. , Li, D. , Fu, X.‐J. , Zhou, Y. , and Liu, M.‐F. (2019). A translation‐activating function of MIWI/piRNA during mouse spermiogenesis. Cell 179: 1566–1581. 31835033 10.1016/j.cell.2019.11.022 PMC8139323

[jipb13422-bib-0013] Deal, R.B. , and Henikoff, S. (2011). The INTACT method for cell type–specific gene expression and chromatin profiling in *Arabidopsis thaliana* . Nat. Protoc. 6: 56–68. 21212783 10.1038/nprot.2010.175 PMC7219316

[jipb13422-bib-0014] Del Toro‐De León, G. , and Köhler, C. (2019). Endosperm‐specific transcriptome analysis by applying the INTACT system. Plant Reprod 32: 55–61. 30588542 10.1007/s00497-018-00356-3

[jipb13422-bib-0015] Dubin, M.J. , Zhang, P. , Meng, D. , Remigereau, M.‐S. , Osborne, E.J. , Paolo Casale, F. , Drewe, P. , Kahles, A. , Jean, G. , Vilhjálmsson, B. , Jagoda, J. , Irez, S. , Voronin, V. , Song, Q. , Long, Q. , Rätsch, G. , Stegle, O. , Clark, R.M. , and Nordborg, M. (2015). DNA methylation in Arabidopsis has a genetic basis and shows evidence of local adaptation. eLife 4: e05255. 25939354 10.7554/eLife.05255 PMC4413256

[jipb13422-bib-0016] Dukowic‐Schulze, S. , Sundararajan, A. , Ramaraj, T. , Kianian, S. , Pawlowski, W.P. , Mudge, J. , and Chen, C. (2016). Novel meiotic miRNAs and indications for a role of PhasiRNAs in Meiosis. Front. Plant Sci. 7: 762. 27313591 10.3389/fpls.2016.00762 PMC4889585

[jipb13422-bib-0017] Dunoyer, P. , Himber, C. , Ruiz‐Ferrer, V. , Alioua, A. , and Voinnet, O. (2007). Intra‐ and intercellular RNA interference in *Arabidopsis thaliana* requires components of the microRNA and heterochromatic silencing pathways. Nat. Genet. 39: 848–856. 17558406 10.1038/ng2081

[jipb13422-bib-0018] Erdmann, R.M. , Hoffmann, A. , Walter, H.‐K. , Wagenknecht, H.‐A. , Groß‐Hardt, R. , and Gehring, M. (2017). Molecular movement in the *Arabidopsis thaliana* female gametophyte. Plant Reprod 30: 141–146. 28695277 10.1007/s00497-017-0304-3 PMC5599461

[jipb13422-bib-0019] Ernst, C. , Odom, D.T. , and Kutter, C. (2017). The emergence of piRNAs against transposon invasion to preserve mammalian genome integrity. Nat. Commun. 8: 1411. 29127279 10.1038/s41467-017-01049-7 PMC5681665

[jipb13422-bib-0020] Fei, Q. , Yang, L. , Liang, W. , Zhang, D. , and Meyers, B.C. (2016). Dynamic changes of small RNAs in rice spikelet development reveal specialized reproductive phasiRNA pathways. J. Exp. Bot. 67: 6037–6049. 27702997 10.1093/jxb/erw361 PMC5100018

[jipb13422-bib-0021] Feng, X. , Zilberman, D. , and Dickinson, H. (2013). A conversation across generations: Soma‐germ cell crosstalk in plants. Dev. Cell 24: 215–225. 23410937 10.1016/j.devcel.2013.01.014

[jipb13422-bib-0022] Figueiredo, D.D. , and Köhler, C. (2016). Bridging the generation gap: Communication between maternal sporophyte, female gametophyte and fertilization products. Curr. Opin. Plant Biol. 29: 16–20. 26658334 10.1016/j.pbi.2015.10.008

[jipb13422-bib-0023] Flores‐Tornero, M. , Proost, S. , Mutwil, M. , Scutt, C.P. , Dresselhaus, T. , and Sprunck, S. (2019). Transcriptomics of manually isolated Amborella trichopoda egg apparatus cells. Plant Reprod. 32: 15–27. 30707279 10.1007/s00497-019-00361-0

[jipb13422-bib-0024] Frost, J.M. , Kim, M.Y. , Park, G.T. , Hsieh, P.‐H. , Nakamura, M. , Lin, S.J.H. , Yoo, H. , Choi, J. , Ikeda, Y. , Kinoshita, T. , Choi, Y. , Zilberman, D. , and Fischer, R.L. (2018). FACT complex is required for DNA demethylation at heterochromatin during reproduction in Arabidopsis. Proc. Natl. Acad. Sci. U.S.A. 115: E4720–E4729. 29712855 10.1073/pnas.1713333115 PMC5960277

[jipb13422-bib-0025] Garcia‐Aguilar, M. , Michaud, C. , Leblanc, O. , and Grimanelli, D. (2010). Inactivation of a DNA methylation pathway in Maize reproductive organs results in apomixis‐like phenotypes. Plant Cell 22: 3249–3267. 21037104 10.1105/tpc.109.072181 PMC2990141

[jipb13422-bib-0026] Gehring, M. (2013). Genomic Imprinting: Insights from plants. Annu. Rev. Genet. 47: 187–208. 24016190 10.1146/annurev-genet-110711-155527

[jipb13422-bib-0027] Gehring, M. , Bubb, K.L. , and Henikoff, S. (2009). Extensive demethylation of repetitive elements during seed development underlies gene imprinting. Science 324: 1447–1451. 19520961 10.1126/science.1171609 PMC2886585

[jipb13422-bib-0028] Gehring, M. , Huh, J.H. , Hsieh, T.‐F. , Penterman, J. , Choi, Y. , Harada, J.J. , Goldberg, R.B. , and Fischer, R.L. (2006). DEMETER DNA glycosylase establishes MEDEA polycomb gene self‐imprinting by allele‐specific demethylation. Cell 124: 495–506. 16469697 10.1016/j.cell.2005.12.034 PMC4106368

[jipb13422-bib-0029] Gómez, J.F. , Talle, B. , and Wilson, Z.A. (2015). Anther and pollen development: A conserved developmental pathway. J. Integr. Plant Biol. 57: 876–891. 26310290 10.1111/jipb.12425 PMC4794635

[jipb13422-bib-0030] Gong, Z. , Morales‐Ruiz, T. , Ariza, R.R. , Roldán‐Arjona, T. , David, L. , and Zhu, J.‐K. (2002). ROS1, a repressor of transcriptional gene silencing in *Arabidopsis*, encodes a DNA glycosylase/lyase. Cell 111: 803–814. 12526807 10.1016/s0092-8674(02)01133-9

[jipb13422-bib-0031] Greenberg, M.V.C. , and Bourc'his, D. (2019). The diverse roles of DNA methylation in mammalian development and disease. Nat. Rev. Mol. Cell Biol. 20: 590–607. 31399642 10.1038/s41580-019-0159-6

[jipb13422-bib-0032] Grossniklaus, U. , Vielle‐Calzada, J.‐P. , Hoeppner, M.A. , and Gagliano, W.B. (1998). Maternal control of embryogenesis by *MEDEA*, a *polycomb* group gene in *Arabidopsis* . Science 280: 446–450. 9545225 10.1126/science.280.5362.446

[jipb13422-bib-0033] Grover, J.W. , Burgess, D. , Kendall, T. , Baten, A. , Pokhrel, S. , King, G.J. , Meyers, B.C. , Freeling, M. , and Mosher, R.A. (2020). Abundant expression of maternal siRNAs is a conserved feature of seed development. Proc. Natl. Acad. Sci. U.S.A. 117: 15305–15315. 32541052 10.1073/pnas.2001332117 PMC7334491

[jipb13422-bib-0034] Hackenberg, D. , and Twell, D. (2019). The evolution and patterning of male gametophyte development. Curr. Top. Dev. Biol. 131: 257–298. 30612620 10.1016/bs.ctdb.2018.10.008

[jipb13422-bib-0035] Han, Y.‐Z. , Huang, B.‐Q. , Zee, S.‐Y. , and Yuan, M. (2000). Symplastic communication between the central cell and the egg apparatus cells in the embryo sac of Torenia fournieri Lind. before and during fertilization. Planta 211: 158–162. 10923717 10.1007/s004250000289

[jipb13422-bib-0036] He, S. , Vickers, M. , Zhang, J. , and Feng, X. (2019). Natural depletion of histone H1 in sex cells causes DNA demethylation, heterochromatin decondensation and transposon activation. eLife 8: e42530. 31135340 10.7554/eLife.42530 PMC6594752

[jipb13422-bib-0037] Hsieh, P.‐H. , He, S. , Buttress, T. , Gao, H. , Couchman, M. , Fischer, R.L. , Zilberman, D. , and Feng, X. (2016). *Arabidopsis* male sexual lineage exhibits more robust maintenance of CG methylation than somatic tissues. Proc. Natl. Acad. Sci. U.S.A. 113: 15132–15137. 27956643 10.1073/pnas.1619074114 PMC5206529

[jipb13422-bib-0038] Hsieh, T.‐F. , Ibarra, C.A. , Silva, P. , Zemach, A. , Eshed‐Williams, L. , Fischer, R.L. , and Zilberman, D. (2009). Genome‐wide demethylation of *Arabidopsis* endosperm. Science 324: 1451–1454. 19520962 10.1126/science.1172417 PMC4044190

[jipb13422-bib-0039] Huang, K. , Wu, X.‐X. , Fang, C.‐L. , Xu, Z.‐G. , Zhang, H.‐W. , Gao, J. , Zhou, C.‐M. , You, L.‐L. , Gu, Z.‐X. , Mu, W.‐H. , Feng, Y. , Wang, J.‐W. , and Zhang, Y. (2021). Pol IV and RDR2: A two‐RNA‐polymerase machine that produces double‐stranded RNA. Science 374: 1579–1586. 34941388 10.1126/science.abj9184

[jipb13422-bib-0040] Ibarra, C.A. , Feng, X. , Schoft, V.K. , Hsieh, T.‐F. , Uzawa, R. , Rodrigues, J.A. , Zemach, A. , Chumak, N. , Machlicova, A. , Nishimura, T. , Rojas, D. , Fischer, R.L. , Tamaru, H. , and Zilberman, D. (2012). Active DNA demethylation in plant companion cells reinforces transposon methylation in Gametes. Science 337: 1360–1364. 22984074 10.1126/science.1224839 PMC4034762

[jipb13422-bib-0041] Ingouff, M. , Selles, B. , Michaud, C. , Vu, T.M. , Berger, F. , Schorn, A.J. , Autran, D. , Van, D.M. , Nowack, M.K. , Martienssen, R.A. , and Grimanelli, D. (2017). Live‐cell analysis of DNA methylation during sexual reproduction in *Arabidopsis* reveals context and sex‐specific dynamics controlled by noncanonical RdDM. Genes Dev. 31: 72–83. 28115468 10.1101/gad.289397.116 PMC5287115

[jipb13422-bib-0042] Jiang, P. , Lian, B. , Liu, C. , Fu, Z. , Shen, Y. , Cheng, Z. , and Qi, Y. (2020). 21‐nt phasiRNAs direct target mRNA cleavage in rice male germ cells. Nat. Commun. 11: 5191. 33060587 10.1038/s41467-020-19034-y PMC7562718

[jipb13422-bib-0043] Johnson, C. , Kasprzewska, A. , Tennessen, K. , Fernandes, J. , Nan, G.‐L. , Walbot, V. , Sundaresan, V. , Vance, V. , and Bowman, L.H. (2009). Clusters and superclusters of phased small RNAs in the developing inflorescence of rice. Genome Res. 19: 1429–1440. 19584097 10.1101/gr.089854.108 PMC2720183

[jipb13422-bib-0044] Kakrana, A. , Mathioni, S.M. , Huang, K. , Hammond, R. , Vandivier, L. , Patel, P. , Arikit, S. , Shevchenko, O. , Harkess, A.E. , Kingham, B. , Gregory, B.D. , Leebens‐Mack, J.H. , and Meyers, B.C. (2018). Plant 24‐nt reproductive phasiRNAs from intramolecular duplex mRNAs in diverse monocots. Genome Res. 28: 1333–1344. 30002159 10.1101/gr.228163.117 PMC6120631

[jipb13422-bib-0045] Kawakatsu, T. , Huang, S.C. , Jupe, F. , Sasaki, E. , Schmitz, R.J. , Urich, M.A. , Castanon, R. , Nery, J.R. , Barragan, C. , He, Y. , Chen, H. , Dubin, M. , Lee, C.‐R. , Wang, C. , Bemm, F. , Becker, C. , O'Neil, R. , O'Malley, R.C. , Quarless, D.X. , Schork, N.J. , Weigel, D. , Nordborg, M. , and Ecker, J.R. (2016a). Epigenomic diversity in a global collection of Arabidopsis thaliana accessions. Cell 166: 492–505. 27419873 10.1016/j.cell.2016.06.044 PMC5172462

[jipb13422-bib-0046] Kawakatsu, T. , Stuart, T. , Valdes, M. , Breakfield, N. , Schmitz, R.J. , Nery, J.R. , Urich, M.A. , Han, X. , Lister, R. , Benfey, P.N. , and Ecker, J.R. (2016b). Unique cell‐type‐specific patterns of DNA methylation in the root meristem. Nat. Plants 2: 16058. 27243651 10.1038/nplants.2016.58 PMC4855458

[jipb13422-bib-0047] Kawashima, T. , and Berger, F. (2014). Epigenetic reprogramming in plant sexual reproduction. Nat. Rev. Genet. 15: 613–624. 25048170 10.1038/nrg3685

[jipb13422-bib-0048] Khouider, S. , Borges, F. , LeBlanc, C. , Ungru, A. , Schnittger, A. , Martienssen, R. , Colot, V. , and Bouyer, D. (2021). Male fertility in *Arabidopsis* requires active DNA demethylation of genes that control pollen tube function. Nat. Commun. 12: 410. 33462227 10.1038/s41467-020-20606-1 PMC7813888

[jipb13422-bib-0049] Kim, M.Y. , Ono, A. , Scholten, S. , Kinoshita, T. , Zilberman, D. , Okamoto, T. , and Fischer, R.L. (2019). DNA demethylation by ROS1a in rice vegetative cells promotes methylation in sperm. Proc. Natl. Acad. Sci. U.S.A. 116: 9652–9657. 31000601 10.1073/pnas.1821435116 PMC6511055

[jipb13422-bib-0050] Kinoshita, T. , Miura, A. , Choi, Y. , Kinoshita, Y. , Cao, X. , Jacobsen, S.E. , Fischer, R.L. , and Kakutani, T. (2004). One‐way control of *FWA* imprinting in *Arabidopsis* endosperm by DNA methylation. Science 303: 521–523. 14631047 10.1126/science.1089835

[jipb13422-bib-0051] Kinoshita, T. , Yadegari, R. , Harada, J.J. , Goldberg, R.B. , and Fischer, R.L. (1999). Imprinting of the *MEDEA* polycomb gene in the *Arabidopsis* endosperm. Plant Cell 11: 1945–1952. 10521524 10.1105/tpc.11.10.1945 PMC144115

[jipb13422-bib-0052] Kiuchi, T. , Koga, H. , Kawamoto, M. , Shoji, K. , Sakai, H. , Arai, Y. , Ishihara, G. , Kawaoka, S. , Sugano, S. , Shimada, T. , Suzuki, Y. , Suzuki, M.G. , and Katsuma, S. (2014). A single female‐specific piRNA is the primary determiner of sex in the silkworm. Nature 509: 633–636. 24828047 10.1038/nature13315

[jipb13422-bib-0053] Kiyosue, T. , Ohad, N. , Yadegari, R. , Hannon, M. , Dinneny, J. , Wells, D. , Katz, A. , Margossian, L. , Harada, J.J. , Goldberg, R.B. , and Fischer, R.L. (1999). Control of fertilization‐independent endosperm development by the MEDEA polycomb gene in Arabidopsis. Proc. Natl. Acad. Sci. U.S.A. 96: 4186–4191. 10097185 10.1073/pnas.96.7.4186 PMC22442

[jipb13422-bib-0054] Komiya, R. , Ohyanagi, H. , Niihama, M. , Watanabe, T. , Nakano, M. , Kurata, N. , and Nonomura, K.‐I. (2014). Rice germline‐specific Argonaute MEL1 protein binds to phasiRNAs generated from more than 700 lincRNAs. Plant J. 78: 385–397. 24635777 10.1111/tpj.12483

[jipb13422-bib-0055] Kuo, H.Y. , Jacobsen, E.L. , Long, Y. , Chen, X. , and Zhai, J. (2017). Characteristics and processing of Pol IV‐dependent transcripts in *Arabidopsis* . J. Genet. Genomics 44: 3–6. 28089091 10.1016/j.jgg.2016.10.009

[jipb13422-bib-0056] Law, J.A. , and Jacobsen, S.E. (2010). Establishing, maintaining and modifying DNA methylation patterns in plants and animals. Nat. Rev. Genet. 11: 204–220. 20142834 10.1038/nrg2719 PMC3034103

[jipb13422-bib-0057] Lewsey, M.G. , Hardcastle, T.J. , Melnyk, C.W. , Molnar, A. , Valli, A. , Urich, M.A. , Nery, J.R. , Baulcombe, D.C. , and Ecker, J.R. (2016). Mobile small RNAs regulate genome‐wide DNA methylation. Proc. Natl. Acad. Sci. U.S.A. 113: E801–E810. 26787884 10.1073/pnas.1515072113 PMC4760824

[jipb13422-bib-0058] Li, C. , Gent, J.I. , Xu, H. , Fu, H. , Russell, S.D. , and Sundaresan, V. (2022). Resetting of the 24‐nt siRNA landscape in rice zygotes. Genome Res. 32: 309–323. 34949668 10.1101/gr.275981.121 PMC8805726

[jipb13422-bib-0059] Li, L. , Wu, W. , Zhao, Y. , and Zheng, B. (2017). A reciprocal inhibition between ARID1 and MET1 in male and female gametes in *Arabidopsis*: MET1 and ARID1 restrict reciprocally in plant gametes. J. Integr. Plant Biol 59: 657–668. 28782297 10.1111/jipb.12573

[jipb13422-bib-0060] Liu, Y. , Teng, C. , Xia, R. , and Meyers, B.C. (2020). PhasiRNAs in plants: Their biogenesis, genic sources, and roles in stress responses, development, and reproduction. Plant Cell 32: 3059–3080. 32817252 10.1105/tpc.20.00335 PMC7534485

[jipb13422-bib-0061] Long, J. , Walker, J. , She, W. , Aldridge, B. , Gao, H. , Deans, S. , Vickers, M. , and Feng, X. (2021). Nurse cell­derived small RNAs define paternal epigenetic inheritance in *Arabidopsis* . Science 373: eabh0556. 34210850 10.1126/science.abh0556

[jipb13422-bib-0062] Luo, M. , Bilodeau, P. , Koltunow, A. , Dennis, E.S. , Peacock, W.J. , and Chaudhury, A.M. (1999). Genes controlling fertilization‐independent seed development in *Arabidopsis thaliana* . Proc. Natl. Acad. Sci. U.S.A. 96: 296‐301. 9874812 10.1073/pnas.96.1.296 PMC15133

[jipb13422-bib-0063] Mamun, E. , Cantrill, L. , Overall, R. , and Sutton, B. (2005). Cellular organisation and differentiation of organelles in pre‐meiotic rice anthers. Cell Biol. Int. 29: 792–802. 16085434 10.1016/j.cellbi.2005.05.009

[jipb13422-bib-0064] Martínez, G. , Panda, K. , Köhler, C. , and Slotkin, R.K. (2016). Silencing in sperm cells is directed by RNA movement from the surrounding nurse cell. Nat. Plants. 2: 16030. 27249563 10.1038/nplants.2016.30

[jipb13422-bib-0065] Matzke, M.A. , and Mosher, R.A. (2014). RNA‐directed DNA methylation: An epigenetic pathway of increasing complexity. Nat. Rev. Genet. 15: 394–408. 24805120 10.1038/nrg3683

[jipb13422-bib-0066] Melnyk, C.W. , Molnar, A. , Bassett, A. , and Baulcombe, D.C. (2011). Mobile 24 nt small RNAs direct transcriptional gene silencing in the root meristems of *Arabidopsis thaliana* . Curr. Biol. 21: 1678–1683. 21962713 10.1016/j.cub.2011.08.065

[jipb13422-bib-0067] Molnar, A. , Melnyk, C.W. , Bassett, A. , Hardcastle, T.J. , Dunn, R. , and Baulcombe, D.C. (2010). Small silencing RNAs in plants are mobile and direct epigenetic modification in recipient cells. Science 328: 872–875. 20413459 10.1126/science.1187959

[jipb13422-bib-0068] Moreno‐Romero, J. , Santos‐González, J. , Hennig, L. , and Köhler, C. (2017). Applying the INTACT method to purify endosperm nuclei and to generate parental‐specific epigenome profiles. Nat. Protoc. 12: 238–254. 28055034 10.1038/nprot.2016.167

[jipb13422-bib-0069] Nan, G.‐L. , Zhai, J. , Arikit, S. , Morrow, D. , Fernandes, J. , Mai, L. , Nguyen, N. , Meyers, B.C. , and Walbot, V. (2016). MS23, a master basic helix‐loop‐helix factor, regulates the specification and development of tapetum in Maize. Development 144: 163–172. 27913638 10.1242/dev.140673

[jipb13422-bib-0070] Olmedo‐Monfil, V. , Durán‐Figueroa, N. , Arteaga‐Vázquez, M. , Demesa‐Arévalo, E. , Autran, D. , Grimanelli, D. , Slotkin, R.K. , Martienssen, R.A. , and Vielle‐Calzada, J.‐P. (2010). Control of female gamete formation by a small RNA pathway in *Arabidopsis* . Nature 464: 628–632. 20208518 10.1038/nature08828 PMC4613780

[jipb13422-bib-0071] Ono, S. , Liu, H. , Tsuda, K. , Fukai, E. , Tanaka, K. , Sasaki, T. , and Nonomura, K.‐I. (2018). EAT1 transcription factor, a non‐cell‐autonomous regulator of pollen production, activates meiotic small RNA biogenesis in rice anther tapetum. PLoS Genet. 14: e1007238. 29432414 10.1371/journal.pgen.1007238 PMC5825165

[jipb13422-bib-0072] Ortega‐Galisteo, A.P. , Morales‐Ruiz, T. , Ariza, R.R. , and Roldán‐Arjona, T. (2008). *Arabidopsis* DEMETER‐LIKE proteins DML2 and DML3 are required for appropriate distribution of DNA methylation marks. Plant Mol. Biol. 67: 671–681. 18493721 10.1007/s11103-008-9346-0

[jipb13422-bib-0073] Ozata, D.M. , Gainetdinov, I. , Zoch, A. , O'Carroll, D. , and Zamore, P.D. (2019). PIWI‐interacting RNAs: Small RNAs with big functions. Nat. Rev. Genet. 20: 89–108. 30446728 10.1038/s41576-018-0073-3

[jipb13422-bib-0074] Parfrey, L.W. , Lahr, D.J.G. , Knoll, A.H. , and Katz, L.A. (2011). Estimating the timing of early eukaryotic diversification with multigene molecular clocks. Proc. Natl. Acad. Sci. U.S.A. 108: 13624–13629. 21810989 10.1073/pnas.1110633108 PMC3158185

[jipb13422-bib-0075] Park, J.‐S. , Frost, J.M. , Park, K. , Ohr, H. , Park, G.T. , Kim, S. , Eom, H. , Lee, I. , Brooks, J.S. , Fischer, R.L. , and Choi, Y. (2017). Control of DEMETER DNA demethylase gene transcription in male and female gamete companion cells in *Arabidopsis thaliana* . Proc. Natl. Acad. Sci. U.S.A. 114: 2078–2083. 28130550 10.1073/pnas.1620592114 PMC5338364

[jipb13422-bib-0076] Park, K. , Frost, J.M. , Adair, A.J. , Kim, D.M. , Yun, H. , Brooks, J.S. , Fischer, R.L. , and Choi, Y. (2016a). Optimized methods for the isolation of *Arabidopsis* female central cells and their nuclei. Mol. Cells 39: 768–775. 27788573 10.14348/molcells.2016.0209 PMC5104886

[jipb13422-bib-0077] Park, K. , Kim, M.Y. , Vickers, M. , Park, J.‐S. , Hyun, Y. , Okamoto, T. , Zilberman, D. , Fischer, R.L. , Feng, X. , Choi, Y. , and Scholten, S. (2016b). DNA demethylation is initiated in the central cells of *Arabidopsis* and rice. Proc. Natl. Acad. Sci. U.S.A. 113: 15138–15143. 27956642 10.1073/pnas.1619047114 PMC5206524

[jipb13422-bib-0078] Penterman, J. , Zilberman, D. , Huh, J.H. , Ballinger, T. , Henikoff, S. , and Fischer, R.L. (2007). DNA demethylation in the *Arabidopsis* genome. Proc. Natl. Acad. Sci. U.S.A. 104: 6752–6757. 17409185 10.1073/pnas.0701861104 PMC1847597

[jipb13422-bib-0079] Pikaard, C.S. , and Scheid O.M. (2014). Epigenetic regulation in plants. Cold Spring Harb. Perspect. Biol. 6: a019315. 25452385 10.1101/cshperspect.a019315 PMC4292151

[jipb13422-bib-0080] Postnikov, Y.V. , and Bustin, M. (2016). Functional interplay between histone H1 and HMG proteins in chromatin. Biochim. Biophys. Acta. (BBA). 1859: 462–467. 26455954 10.1016/j.bbagrm.2015.10.006 PMC4852864

[jipb13422-bib-0081] Rojas‐Ríos, P. , and Simonelig, M. (2018). piRNAs and PIWI proteins: Regulators of gene expression in development and stem cells. Development 145: dev161786. 30194260 10.1242/dev.161786

[jipb13422-bib-0082] Sager, R. , and Lee, J.‐Y. (2014). Plasmodesmata in integrated cell signalling: Insights from development and environmental signals and stresses. J. Exp. Bot. 65: 6337–6358. 25262225 10.1093/jxb/eru365 PMC4303807

[jipb13422-bib-0083] Santos, M.R. , Bispo, C. , and Becker, J.D. (2017). Isolation of *Arabidopsis* pollen, sperm cells, and vegetative nuclei by fluorescence‐activated cell sorting (FACS). Methods Mol. Biol. 1669: 193–210. 28936660 10.1007/978-1-4939-7286-9_16

[jipb13422-bib-0084] Schmidt, A. , Schmid, M.W. , and Grossniklaus, U. (2015). Plant germline formation: Common concepts and developmental flexibility in sexual and asexual reproduction. Development 142: 229–241. 25564620 10.1242/dev.102103

[jipb13422-bib-0085] Schmidt, A. , Wuest, S.E. , Vijverberg, K. , Baroux, C. , Kleen, D. , and Grossniklaus, U. (2011). Transcriptome analysis of the *Arabidopsis* megaspore mother cell uncovers the importance of RNA helicases for plant germline development. PLoS Biol. 9: e1001155. 21949639 10.1371/journal.pbio.1001155 PMC3176755

[jipb13422-bib-0086] Schoft, V.K. , Chumak, N. , Choi, Y. , Hannon, M. , Garcia‐Aguilar, M. , Machlicova, A. , Slusarz, L. , Mosiolek, M. , Park, J.‐S. , Park, G.T. , Fischer, R.L. , and Tamaru, H. (2011). Function of the DEMETER DNA glycosylase in the *Arabidopsis thaliana* male gametophyte. Proc. Natl. Acad. Sci. U.S.A. 108: 8042–8047. 21518889 10.1073/pnas.1105117108 PMC3093457

[jipb13422-bib-0087] Seisenberger, S. , Peat, J.R. , Hore, T.A. , Santos, F. , Dean, W. , and Reik, W. (2013). Reprogramming DNA methylation in the mammalian life cycle: Building and breaking epigenetic barriers. Philos. Trans. R. Soc. Lond. B. Biol. Sci. 368: 20110330. 23166394 10.1098/rstb.2011.0330 PMC3539359

[jipb13422-bib-0088] Slotkin, R.K. , Vaughn, M. , Borges, F. , Tanurdžić, M. , Becker, J.D. , Feijó, J.A. , and Martienssen, R.A. (2009). Epigenetic reprogramming and small RNA silencing of transposable elements in pollen. Cell 136: 461–472. 19203581 10.1016/j.cell.2008.12.038 PMC2661848

[jipb13422-bib-0089] Smallwood, S.A. , Lee, H.J. , Angermueller, C. , Krueger, F. , Saadeh, H. , Peat, J. , Andrews, S.R. , Stegle, O. , Reik, W. , and Kelsey, G. (2014). Single‐cell genome‐wide bisulfite sequencing for assessing epigenetic heterogeneity. Nat. Methods 11: 817–820. 25042786 10.1038/nmeth.3035 PMC4117646

[jipb13422-bib-0090] Smith, L.M. , Pontes, O. , Searle, I. , Yelina, N. , Yousafzai, F.K. , Herr, A.J. , Pikaard, C.S. , and Baulcombe, D.C. (2007). An SNF2 protein associated with nuclear RNA silencing and the spread of a silencing signal between cells in *Arabidopsis* . Plant Cell 19: 1507–1521. 17526749 10.1105/tpc.107.051540 PMC1913737

[jipb13422-bib-0091] Smith, Z.D. , and Meissner, A. (2013). DNA methylation: Roles in mammalian development. Nat. Rev. Genet. 14: 204–220. 23400093 10.1038/nrg3354

[jipb13422-bib-0092] Song, X. , Li, P. , Zhai, J. , Zhou, M. , Ma, L. , Liu, B. , Jeong, D.‐H. , Nakano, M. , Cao, S. , Liu, C. , Chu, C. , Wang, X.‐J. , Green, P.J. , Meyers, B.C. , and Cao, X. (2012). Roles of DCL4 and DCL3b in rice phased small RNA biogenesis: Phased small RNA clusters biogenesis in rice. Plant J. 69: 462–474. 21973320 10.1111/j.1365-313X.2011.04805.x

[jipb13422-bib-0093] Sprunck, S. , Baumann, U. , Edwards, K. , Langridge, P. , and Dresselhaus, T. (2005). The transcript composition of egg cells changes significantly following fertilization in wheat (*Triticum aestivum* L.): Transcriptome of wheat eggs and proembryos. Plant J. 41: 660–672. 15703054 10.1111/j.1365-313X.2005.02332.x

[jipb13422-bib-0094] Stewart, K.R. , Veselovska, L. , and Kelsey, G. (2016). Establishment and functions of DNA methylation in the germline. Epigenomics 8: 1399–1413. 27659720 10.2217/epi-2016-0056 PMC5066131

[jipb13422-bib-0095] Su, Z. , Wang, N. , Hou, Z. , Li, B. , Li, D. , Liu, Y. , Cai, H. , Qin, Y. , and Chen, X. (2020). Regulation of female germline specification via small RNA mobility in *Arabidopsis* . Plant Cell 32: 2842–2854. 32703817 10.1105/tpc.20.00126 PMC7474286

[jipb13422-bib-0096] Su, Z. , Zhao, L. , Zhao, Y. , Li, S. , Won, S. , Cai, H. , Wang, L. , Li, Z. , Chen, P. , Qin, Y. , and Chen, X. (2017). The THO complex non‐cell‐autonomously represses female germline specification through the TAS3‐ARF3 Module. Curr. Biol. 27: 1597–1609. 28552357 10.1016/j.cub.2017.05.021 PMC5544534

[jipb13422-bib-0097] Tang, W.W.C. , Kobayashi, T. , Irie, N. , Dietmann, S. , and Surani, M.A. (2016). Specification and epigenetic programming of the human germ line. Nat. Rev. Genet. 17: 585–600. 27573372 10.1038/nrg.2016.88

[jipb13422-bib-0098] Teng, C. , Zhang, H. , Hammond, R. , Huang, K. , Meyers, B.C. , and Walbot, V. (2020). Dicer‐like 5 deficiency confers temperature‐sensitive male sterility in maize. Nat. Commun. 11: 2912. 32518237 10.1038/s41467-020-16634-6 PMC7283321

[jipb13422-bib-0099] Tóth, K.F. , Pezic, D. , Stuwe, E. , and Webster, A. (2016). The piRNA pathway guards the germline genome against transposable elements. Adv. Exp. Med. Biol. 886: 51–77. 26659487 10.1007/978-94-017-7417-8_4 PMC4991928

[jipb13422-bib-0100] Vielle‐Calzada, J.‐P. (2017). Linking stem cells to germ cells. Science 356: 378–379. 28450598 10.1126/science.aan2734

[jipb13422-bib-0101] Walker, J. , Gao, H. , Zhang, J. , Aldridge, B. , Vickers, M. , Higgins, J.D. , and Feng, X. (2018). Sexual‐lineage‐specific DNA methylation regulates meiosis in *Arabidopsis* . Nat. Genet. 50: 130–137. 29255257 10.1038/s41588-017-0008-5 PMC7611288

[jipb13422-bib-0102] Watson, J.M. , Platzer, A. , Kazda, A. , Akimcheva, S. , Valuchova, S. , Nizhynska, V. , Nordborg, M. , and Riha, K. (2016). Germline replications and somatic mutation accumulation are independent of vegetative life span in Arabidopsis. Proc. Natl. Acad. Sci. U.S.A. 113: 12226–12231. 27729523 10.1073/pnas.1609686113 PMC5087024

[jipb13422-bib-0103] Wendte, J.M. , and Pikaard, C.S. (2017). The RNAs of RNA‐directed DNA methylation. Biochim. Biophys. Acta, Gene Regul. Mech. 1860: 140–148. 27521981 10.1016/j.bbagrm.2016.08.004 PMC5203809

[jipb13422-bib-0104] Xia, R. , Chen, C. , Pokhrel, S. , Ma, W. , Huang, K. , Patel, P. , Wang, F. , Xu, J. , Liu, Z. , Li, J. , and Meyers, B.C. (2019). 24‐nt reproductive phasiRNAs are broadly present in angiosperms. Nat. Commun. 10: 627. 30733503 10.1038/s41467-019-08543-0 PMC6367383

[jipb13422-bib-0105] Xu, Q. , Wu, L. , Luo, Z. , Zhang, M. , Lai, J. , Li, L. , Springer, N.M. , and Li, Q. (2022). DNA demethylation affects imprinted gene expression in maize endosperm. Genome Biol. 23: 77. 35264226 10.1186/s13059-022-02641-x PMC8905802

[jipb13422-bib-0106] Zemach, A. , Kim, M.Y. , Silva, P. , Rodrigues, J.A. , Dotson, B. , Brooks, M.D. , and Zilberman, D. (2010). Local DNA hypomethylation activates genes in rice endosperm. Proc. Natl. Acad. Sci. U.S.A. 107: 18729–18734. 20937895 10.1073/pnas.1009695107 PMC2972920

[jipb13422-bib-0107] Zemach, A. , and Zilberman, D. (2010). Evolution of eukaryotic DNA methylation and the pursuit of safer sex. Curr. Biol. 20: R780–R785. 20833323 10.1016/j.cub.2010.07.007

[jipb13422-bib-0108] Zhai, J. , Zhang, H. , Arikit, S. , Huang, K. , Nan, G.‐L. , Walbot, V. , and Meyers, B.C. (2015). Spatiotemporally dynamic, cell‐type–dependent premeiotic and meiotic phasiRNAs in maize anthers. Proc. Natl. Acad. Sci. U.S.A. 112: 3146–3151. 25713378 10.1073/pnas.1418918112 PMC4364226

[jipb13422-bib-0109] Zhang, H. , Lang, Z. , and Zhu, J.‐K. (2018). Dynamics and function of DNA methylation in plants. Nat. Rev. Mol. Cell Biol. 19: 489–506. 29784956 10.1038/s41580-018-0016-z

[jipb13422-bib-0110] Zhang, M. , Ma, X. , Wang, C. , Li, Q. , Meyers, B.C. , Springer, N.M. , and Walbot, V. (2021). CHH DNA methylation increases at 24‐ *PHAS* loci depend on 24‐nt phased small interfering RNAs in maize meiotic anthers. New Phytol. 229: 2984–2997. 33135165 10.1111/nph.17060

[jipb13422-bib-0111] Zhao, P. , Zhou, X. , Shen, K. , Liu, Z. , Cheng, T. , Liu, D. , Cheng, Y. , Peng, X. , and Sun, M. (2019). Two‐step maternal‐to‐zygotic transition with two‐phase parental genome contributions. Dev. Cell 49 : 882–893. 10.1016/j.devcel.2019.04.016 31080059

[jipb13422-bib-0112] Zheng, X. , and Gehring, M. (2019). Low‐input chromatin profiling in *Arabidopsis* endosperm using CUT&RUN. Plant Reprod. 32: 63–75. 30719569 10.1007/s00497-018-00358-1

[jipb13422-bib-0113] Zhou, M. , Coruh, C. , Xu, G. , Martins, L.M. , Bourbousse, C. , Lambolez, A. , and Law, J.A. (2022a). The CLASSY family controls tissue‐specific DNA methylation patterns in *Arabidopsis* . Nat. Commun. 13: 244. 35017514 10.1038/s41467-021-27690-x PMC8752594

[jipb13422-bib-0114] Zhou, M. , Palanca, A.M.S. , and Law, J.A. (2018). Locus‐specific control of the de novo DNA methylation pathway in *Arabidopsis* by the CLASSY family. Nat. Genet. 50: 865–873. 29736015 10.1038/s41588-018-0115-y PMC6317521

[jipb13422-bib-0115] Zhou, X. , Huang, K. , Teng, C. , Abdelgawad, A. , Batish, M. , Meyers, B.C. , and Walbot, V. (2022b). 24‐nt phasiRNAs move from tapetal to meiotic cells in maize anthers. New Phytol. 235: 488–501. 35451503 10.1111/nph.18167

[jipb13422-bib-0116] Zhu, J.‐K. (2009). Active DNA demethylation mediated by DNA glycosylases. Annu. Rev. Genet. 43: 143–166. 19659441 10.1146/annurev-genet-102108-134205 PMC3137514

